# Prevention of Poor Physical and Mental Health through the Green Social Prescribing Opening Doors to the Outdoors Programme: A Social Return on Investment Analysis

**DOI:** 10.3390/ijerph20126111

**Published:** 2023-06-12

**Authors:** Abraham Makanjuola, Mary Lynch, Ned Hartfiel, Andrew Cuthbert, Rhiannon Tudor Edwards

**Affiliations:** 1 Centre for Health Economics and Medicines Evaluation (CHEME), College of Health and Behavioural Sciences, Bangor University, Bangor LL57 2PZ, UK; ned.hartfiel@bangor.ac.uk (N.H.); r.t.edwards@bangor.ac.uk (R.T.E.); 2 Faculty of Nursing and Midwifery, Royal College of Surgeons in Ireland, D02 YN77 Dublin, Ireland; maryalynch@rcsi.ie; 3 School of Medicine Cardiff, College of Biomedical and Life Sciences, Cardiff University, Cardiff CF14 4EP, UK; cuthberta@cardiff.ac.uk

**Keywords:** health economics, prevention, social return on investment (SROI), social cost–benefit analysis, green social prescribing, mental health, wellbeing, physical activity

## Abstract

There is growing interest in green social prescribing and connecting with nature-based activities to promote social cohesion along with improving levels of health, wealth and well-being. The Outdoor Partnership is a third sector organisation based in North Wales offering nature based social prescribing interventions. Individuals experiencing poor mental health and wellbeing are referred from GPs, community mental health services, and third sector organisations to the ‘Opening the Doors to the Outdoors’ (ODO) programme which is a 12-week outdoor walking and climbing green prescribing intervention. The purpose of the ODO programme is to provide a supportive environment to increase levels of physical activity among participants leading to improvements in overall health and mental wellbeing while promoting socialisation among peers. In this evaluation of a preventative green social prescribing intervention, a mixed method social return on investment (SROI) approach used quantitative and qualitative data from ODO participants. Data collection took place from April 2022–November 2022. Mental wellbeing data was collected at baseline and at 12 weeks using the Short Warwick Edinburgh Mental Wellbeing Scale, a social trust question, an overall health question, and the International Physical Activity Questionnaire- short form. Baseline and follow-up data was available for 52 ODO participants. Results indicate that for every £1 invested in the ODO programme, social values ranging from £4.90 to £5.36 were generated.

## 1. Introduction

### 1.1. Background 

Increased levels of physical activity are associated with higher physical and mental wellbeing, quality of life, reductions in all-cause mortality and improved life expectancy [1,2,3]. Preventive care spending in the UK grew by 40.8% to £15.7 billion in 2020 as a result of the COVID-19 pandemic [4]. Although the UK Chief Medical Officer advises that adults do a minimum of 150 min per week of moderate intensity exercise, 34% of men and 42% of women in the UK are not active enough [5,6]. In addition to physical inactivity, the percentage of people reporting depression in the UK rose from 10% pre-pandemic to 17% by July 2021 [7]. In 2022, the NHS in England injected £4 million to integrate green social prescribing into local communities to improve physical activity, combat low mental wellbeing and reduce health inequalities [8].

An example of green social prescribing is the Opening the Doors to the Outdoors programme (ODO). The ODO programme specifically aims to address the inactivity levels of people experiencing poor mental wellbeing. People who experience enduring low mental wellbeing have a shorter life expectancy, often due to a sedentary lifestyle and neglect of their physical and mental health [9]. ODO provides people with the opportunity to build motivation, confidence, self-esteem, physical fitness and quality of life. It provides socialisation opportunities in a supportive environment for individuals, experiencing low mental wellbeing.

The ODO programme is a complex intervention which refers to a programme that has multiple components that span a number of different skills and expertise, these include the skills required for delivery and participation in the intervention, the number of people or groups targeted, and the flexibility of the intervention itself [10]. The Medical Research Council (MRC) framework for developing and evaluating complex interventions recommends asking questions that pertain to outlining intervention impact, value assessment relative to its inputs, how it contributes to change and how the evidence can inform policy and decision making [11]. Investment into preventative complex health and social care interventions can have a long-term social benefit on wider society in the UK. A recent systematic review suggests that local and national preventative public health interventions in the UK are highly cost-saving [12].

### 1.2. The Outdoor Partnership Programme

In February 2022, the Life Sciences Hub Wales at Cardiff University sponsored a formal study to investigate the social return on investment generated from The Outdoor Partnership’s ‘Opening Doors to the Outdoors’ (ODO) programme. The Outdoor Partnership sought independent evidence of effectiveness and social value to provide legitimacy of the ODO programme as a viable social prescribing referral option. This study was sponsored by the Accelerate Wales healthcare R&D innovation programme, part funded by the Welsh Government’s European Regional Development Fund—Economic Strand. The study received ethical approval from Bangor University and was conducted by researchers at the Social Value Hub at the Centre for Health Economics and Medicines Evaluation (CHEME), Bangor University.

The Outdoor Partnership supports people living in North Wales to increase physical activity and improve mental wellbeing by encouraging grassroots participation in activities such as outdoor walking and climbing. The Outdoor Partnership’s ODO programme is a four-year project funded by the ‘Healthy and Active Fund’. The Healthy and Active Fund is a £5 million investment from the Welsh Government in projects and organisations that support individuals with inactive lifestyles become more active [9].

The ODO programme is a 12-week intervention consisting of one 4-h session per week. The ODO sessions focus on either outdoor walking or climbing activities. Each session over the 12 weeks of the programme includes time for socialisation, either as an outdoor picnic or café visit. The ODO instructors encourage participants to connect with one another and develop friendships. The ODO programme occurs in seven sites across North Wales: six of the sites offer walking interventions (Anglesey, Caernarfon, Dolgellau, Holyhead, Porthmadog, and Wrexham) and one site offers a climbing intervention located in Anglesey. Participants are referred to ODO programmes by GPs, community mental health teams (CMHTs), job centres, third sector organisations, and substance misuse rehabilitation centres such as Adferiad Recovery and Penryn House.

At the start of the 12-week ODO programme, participants had an initial meeting to discuss their current level of physical activity and their willingness to engage in walking outdoors in nature in a guided group or undertaking climbing activities with trained instructors. Risk assessments of participant’s abilities to engage with the ODO programme were conducted by the trained instructors locally at the beginning of the programme.

#### 1.2.1. Walking Groups

Each weekly session focused on outdoor walking, hill and mountain skills, and social connection. During the 12-session intervention, four sessions focused on the Outdoor Partnership’s ‘Hill and Mountain Skills’ course which provided participants with the skills and knowledge to be independent walkers in future. The routes taken by walking groups changed weekly to avoid repetition. ODO instructors progressed the difficulty of the walks by gauging the abilities of the participants.

#### 1.2.2. Climbing Groups

Each weekly session focused on climbing skills and social connection. Although climbing was the major activity, alternative activities included outdoor climbing depending on the ability level of the group. Climbing skills included learning about climbing equipment and how to use it correctly, low intensity traversing, low intensity bouldering, top rope climbing, abseiling, and climbing efficiency. A café visit at the end of the session was offered to encourage social connection.

The purpose of this SROI evaluation of the ODO programme was to examine the social cost-benefit analysis (SCBA), and the associated social value that is produced based on calculation of inputs, outputs and agreed outcomes of this green prescribing intervention. A value ratio is generated and associated with the cost per participant with the social value generated per participant. The SROI analysis of the ODO programme followed the six key stages encouraged for a robust evaluation [13] and adhering to the guidance advocated in the HM Treasury Green Book [14].

## 2. Methods

### 2.1. Social Return on Investment Methodology

The Cost Benefit Analysis (CBA) approach to evaluating public health inventions is recommend by The National Institute for Health and Care Excellence (NICE) and SCBA is endorsed in the HM Treasury Green Book for measuring the impact of interventions on health and wellbeing [14,15]. SROI is a practical mixed method approach to value pertinent costs, intended and unintended outcomes of a public health intervention taking a bottom up approach by engaging with stakeholders from the beginning to identify inputs, agree outputs and outcomes to establish impact [16,17,18].

Undertaking an SROI analysis facilitates the capturing of value and social value generated as a result of a public health intervention and places monetary values on agreed outcomes. This enables organisations to demonstrate the amount of social value that is created based on the amount that is invested in delivering the public health intervention. The SROI deliberates the agreed outcomes that are applicable and important to stakeholders and then assigns monetary values to these outcomes. In this evaluation of the ODO programme outcomes were agreed to be increased levels of overall health, mental wellbeing, physical activity, and social trust and do not have a market value associated. The SROI ratio is estimated by means of wellbeing valuation, the social value of related agreed outcomes was compared with the total costs of delivering the intervention.

In this evaluation of the ODO programme, the wellbeing valuation was generated using two social value calculators: the social value calculator resulting from the Social Value Bank (SVB), and the Mental Health Social Value Calculator derived from the Short Warwick Edinburgh Mental Wellbeing Scale (SWEMWBS). These are strong methods for assessing the monetary value of the agreed outcomes of the intervention with no associated market values. In addition, the Mental Health Social Value Calculator was applied to monetise mental wellbeing from SWEMWBS scores gathered for the evaluation [19] as shown in (Table 1).

### 2.2. Identifying Stakeholders

In this SROI evaluation the main stakeholders were the participants who engaged and experienced the ODO programme, along with the National Health Service (NHS) which could have benefitted from a reduction in mental health service resource use as a result of ODO participants engaging with the green prescribing programme. The agreed outcome data and mental health service resource use data was collected from ODO participants at the beginning and completion of the programme.

Eligibility for inclusion in this evaluation was based on the following criteria: All participants were aged 18 years and above, were referred to the programme as they were experiencing a physical, mental or social issue and could benefit from participating in the ODO programme. All participants were required to be able to speak English or Welsh and have the mental capacity to be able to reflect on their own wellbeing and identify benefits of participating in the ODO programme.

In this evaluation the only participants included in the SROI analysis were ODO participants who completed a baseline and follow-up questionnaire which captured demographic information, baseline and follow-up outcomes for overall health, physical activity, mental wellbeing, social trust, and mental health service resource use.

### 2.3. Developing a Theory of Change

**Figure 1:**
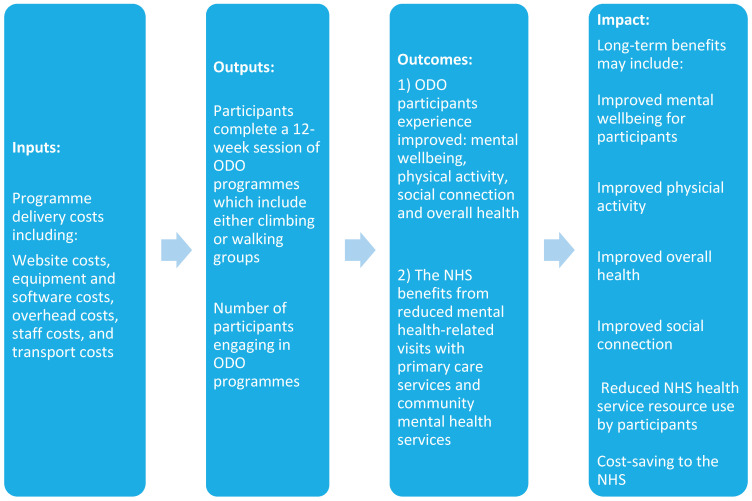
Theory of change model.

A key step in the SROI evaluation of the ODO programme was the creation of the theory of change model (TCM). This TCM was developed by establishing inputs, agreeing outputs and outcomes and the anticipated impact and changes experienced by participants in the ODO programme (Figure 1).

### 2.4. Calculating Inputs

Total costs for ODO programmes included website costs, equipment and software costs, overhead costs, staffing costs, session costs and transport costs. Total annual costs for the ODO programme were 17% of the total annual costs of The Outdoor Partnership.

#### 2.4.1. Website Costs

Website costs included website domain hosting, and monthly costs for secure payment systems.

#### 2.4.2. Equipment and Software Costs

Equipment and software costs included the cost of the Health and Wellbeing Officer’s (HWO) laptop, mobile phone handset, mobile phone contract, and spare waterproof materials for participants (waterproof tops and bottoms, walking poles and walking boots). Annual mobile phone and laptop costs were based on five years that the laptop and mobile phone were expected to perform under heavy usage.

#### 2.4.3. Overhead Costs

Overhead costs included ongoing operation costs such as insurance, accounting and payroll, and rent for the office. The office rent included the cost of electricity, heating, internet, maintenance, office supplies, and telephone bills. Insurance covered both general liability insurance and professional liability insurance.

#### 2.4.4. Staff Costs

Staff costs included 40% FTE of a Health and Wellbeing Officer (HWO) and 40% FTE of a Programme Support Officer (PSO). The Health and Wellbeing Officer was responsible for the day-to-day operations of all Outdoor Partnerships Projects. This role included administrative duties such as developing referrals, communicating with referral organisations, booking participants onto the programme, following up with participants after completing the programme, weekly coordination with instructors and weekly social media updates. The Programme Support Officer was responsible for overall project management of The Outdoor Partnership organisation.

#### 2.4.5. ODO Programme Session Costs

Session costs for the walking groups included the cost of the instructor and expenses for the café visit for participants. Session costs for the climbing group included the cost of the instructor, admission fees for participants, equipment hire and expenses for the café visit for participants post session.

#### 2.4.6. Transport Costs

Transport costs were the costs for the participants to travel to and from outdoor venues. Travel costs included monthly van lease costs, vehicle insurance, road tax, average petrol/mileage costs. Before the COVID-19 pandemic, referral organisations were responsible for the cost of transporting participants to and from their sessions. However, the Outdoor Partnership assumed responsibility for transport of participants utilising its services during and after the pandemic. 

### 2.5. Evidencing and Valuing Outcomes

Outcome measures included the Short Warwick Edinburgh Mental Wellbeing Scale (SWEMWBS), International Physical Activity Questionnaire—Short Form (IPAQ-SF), and the New Economics Foundation (NEF) social trust question, and an overall health question. In addition, a client service resource inventory (CSRI) form was created to measure participant engagement with NHS mental health services. The HACT social value calculator v.4 was used to monetise the outcomes of increased overall health, social trust, and physical activity. The HACT mental health social value calculator v.1 was used to monetise the outcome of mental wellbeing. Qualitative data was collected from interviews (*n* = 6).

#### 2.5.1. Questionnaires

This SROI evaluation included 52 participants from different ODO sites across Wales who completed a baseline questionnaire and a follow-up questionnaire. The questionnaires included demographic information, pre and post intervention outcome measures, health service resource use and additional questions were asked pertaining to the participant’s experience of the ODO programme. The SROI analysis, in this evaluation only includes data from participants who completed the 12-week ODO programme along with completing pre and post programme questionnaires.

Questionnaire data was analysed to determine the number of participants who improved, stayed the same, or worsened for each outcome. The decision to measure and value the four outcomes below was piloted and co-produced by The Outdoor Partnership, the Bangor University research team, former ODO participants, and recommendations from a previous evaluation of The Outdoor Partnership [20].

1. Short Warwick–Edinburgh Mental Wellbeing Scale (SWEMWBS)

The SWEMWBS tool was utilised in this SROI evaluation to assess the mental wellbeing among ODO participants [19]. The SWEMWBS tool contained seven positively worded statements with five response groupings linked with the characteristics of positive mental health and with a scoring range from 7 to 35 [21].

2. The New Economics Foundation Social Trust Question NEF Social Trust Question

Stakeholder engagement in this SROI evaluation established that social trust and relying on others was a key outcome associated with the ODO green prescribing intervention [22]. The inclusion of a social trust question within the evaluation survey was to ascertain the level of trust ODO participants experienced as a result of engaging with the programme [15] with scores ranging from 0 to 10.

3. Overall health question

At follow-up, participants were asked to respond to three statements about what has changed for them due to the programme: ‘I feel fitter’; ‘I feel like I am able to take better care of myself’; ‘My overall physical health has improved’. Overall scores for each statement ranged from 1 to 4, where 1 = little change, 2 = some change, 3 = quite a lot of change and 4 = a lot of change.

4. The International Physical Activity Questionnaire—Short Form (IPAQ—SF)

Participants were asked: “During the last 7 days, on how many days did you do vigorous physical activities like heavy lifting, digging, aerobics, or fast bicycling? Overall scores can range from 0 to 7. The IPAQ-SF is a reliable questionnaire when specifically applied to monitoring levels of physical activity among adults aged 18–65 [23,24].

#### 2.5.2. Client Service Receipt Inventory (CSRI)

As part of this SROI evaluation a CSRI form was adapted to take account of participants use of NHS resources three-months prior to and three-months during the ODO green prescribing intervention. This form was integrated into the SROI evaluation to record the number of mental health-related visits with primary care health professionals such as GPs, nurses as well as the community mental health team [25].

#### 2.5.3. Interviews

The sampling approach applied in this evaluation was a convenience sample of six individuals referred from mental health services to understand participants’ experience of the ODO programme. All participants were experiencing chronic low mood and depression which impacted on lack of engagement in physical activity and decreased socialisation.

Facilitated by a Bangor University researcher, informed consent was obtained from participants prior to being interviewed. Interviews were audio-recorded and transcribed. The interviews were undertaken between August and November 2022 with individuals who had completed the ODO programme. The interviews took place online at a time and in an environment that suited the interviewee and lasted up to 30 min. The interviews were conducted by the main researcher (AM) who is an experienced mixed method researcher.

The semi-structured interviews confirmed the theory of change which was established to understand the inputs to the delivery of the ODO programme, outputs, the expected outcome benefits and agreed anticipated impacts of participating in the ODO programme.

Thematic narrative analysis was conducted on all data gathered from the six interviews to catalogue the findings under key themes of social connection, mental wellbeing, improved over all health and physical activity [26,27]. The interpretation of the data was conducted by (AM and ML) to ensure rigor.

#### 2.5.4. Wellbeing Valuation Using the Social Value Calculator

In this SROI evaluation once the data was computed the wellbeing valuation was utilised to allocate a monetary value on the amount of change experienced by ODO participants. This required applying the Social Value Calculator and values from the HACT Social Value Bank (SVB), which is comprised of operationally consistent and robust social values in the assessment of social value associated with ODO programme.

#### 2.5.5. Wellbeing Valuation Using the Mental Health Social Value Calculator

The Mental Health Social Value Calculator was utilised for baseline and follow-up SWEMWBS scores for each of the ODO participants and were verified and values assigned [19].

##### Applying Mental Health Social Value Calculator

**Figure 2:**
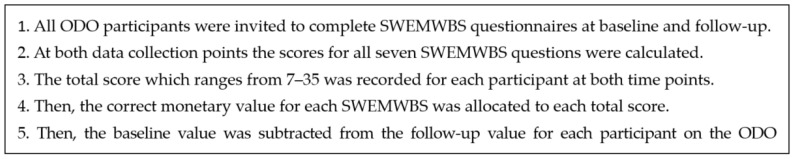
Calculating social value using SWEMWBS.

The Mental Health Social Value Calculator, used the five steps to estimate the social value [19] baseline and follow-up SWEMWBS scores for each ODO participant and were documented and values allotted (Figure 2):

### 2.6. Establishing Impact

When conducting an SROI evaluation it is imperative not to over-claim within the results and therefore, deadweight, attribution and displacement are considered. Deadweight is considered to acknowledge that there is a likelihood that a proportion of the agreed outcomes could have occurred regardless of the ODO programme. In this SROI evaluation, the follow-up questionnaire asked participants to consider what positive change they experienced due to participating in the ODO programme and contemplate what could have happened nonetheless. It is important to take account of levels of attribution and that a percentage of the agreed outcomes could have occurred due to factors other than the ODO programme. In the follow-up questionnaire displacement was taken into account to reflect on whether ODO participants had to give up any other activities that potentially could have contributed to their wellbeing. Finally, to circumvent over-claiming when utilising the HACT Mental Health Social Value Calculator, a 27% standard deadweight percentage for health outcomes was subtracted from the SWEMWBS values [28].

### 2.7. Calculating the SROI Ratio

Using the social value calculator and mental health social value calculator, wellbeing valuation generated SROI ratios that compared the social value of relevant outcomes with the total costs (Equation (1)).

(1) $$\text{SROI ratio}=\frac{\text{Social value of ODO participant outcomes}}{\text{Cost of delivering ODO programme}}$$

## 3. Results

During this 8-month evaluation conducted between April 2022 and November 2022, 75 participants from different ODO sites across Wales completed a baseline questionnaire at the start of their programme and 52 participants (69% response rate) completed a follow-up questionnaire at the end of their programme. Questionnaires were co-produced by The Outdoor Partnership, Bangor University research team and previous participants of Outdoor Partnership programmes.

Of the 52 participants who completed the follow-up questionnaire (Table 2):

- 96% were aged 18 to 64
- 64% were male, 34% women and 2% transgender
- 94% were white British, 4% mixed ethnicity and 2% Asian

### 3.1. Costs

The total value of the inputs for the ODO Programme were GBP 74,129 per year. The largest costs to run the ODO programme were session and staffing costs which were valued at GBP 36,065 per year and GBP 34,023 per year, respectively. The total costs per participant for the ODO programme was GBP 706 per person per year (Table 3).

### 3.2. Outcomes Using the Social Value Calculator

In this evaluation, to ascertain if the agreed outcomes were achieved and to monetise these improvements, the Social Value Calculator was used to provide the value estimates in GBP (£) for changes in the key outcomes of social trust, good overall health, and increases in physical activity by engaging in walking and climbing activities. Applying the Social Value Bank (SVB) to examine social trust for improved social trust among ODO participants with comparisons calculated based on pre and post questionnaire values. The SVB value for improvements in social trust was estimated at £3753 per person per year, which is the value assigned to ‘feeling a sense of belonging to neighbourhood’. This is indicated by a 10% change in the NEF Social Trust baseline and follow-up responses.

- To examine the value of good overall health, among participants’ values pre and post evaluations were calculated for improved overall health. The SVB value was estimated for improvements in good overall health at £20,141 per person per year, which was indicated by an improvement of 50% or more, based on pre and post questionnaires.
- To understand the value connected with improvements in Physical Activity among ODO participants, the SVB value estimate indicated an increased value associated with increased physical activity and is estimated at £5281 per person per year, which was the value assigned to a 60-min change per week in regular walking. All value estimates for improvements are outlined in Table 4.

**Table 4:** Quantity of outcomes and total social value.

Outcomes	Indicators	Quantity Improved	Financial Value	Total Social Value for Participants	Social Value per Participant
**Social trust**	NEF social trust question	27/47 reported an increase of 10% or more	£3753 per year for feeling a sense of belonging to neighbourhood	£101,331	£2156 (*n* = 47)
**Good overall health**	Overall health question	19/35 reported an improvement of 50% or more	£20,141 per person per year for significant improvement in good overall health	£382,679	£10,934 (*n* = 35)
**Physical activity** **(walking)**	IPAQ-SF	22/50 reported an increase of 60 min or more per week	£5281 per year for walking	£116,182	£2324 (*n* = 50)
				**£597,883**	**£15,414**

#### Total Social Value from the Social Value Calculator v.4

When deadweight, attribution and displacement were considered, the total social value for participants experiencing better overall health, social trust and physical activity was £134,653 and the total social value per participant was £3458 (Table 5).

### 3.3. Outcomes Using the Mental Health Social Value Calculator

Using the five-step methodology, the total social value using SWEMWBS, was £3788 per participant for ODO participants (Table 6).

### 3.4. Outcomes from the CSRI Questionnaire

Questionnaires completed by participants measured mental health service resource use by comparing the number of mental health-related visits to NHS professionals. The researchers did not ask about GP attendances given that primary care is the gateway to secondary care to mental health services i.e., community psychiatric services. In the case of an incomplete referral, participants would return to the GP. This could happen multiple times before correct referral is achieved and would not be truly indicative of the use of health resources.

Participants were asked about the number of mental health-related visits for two different time periods—three months preceding their ODO programme, and three months during their ODO programme. The results showed that during the ODO programme, participants reported five more visits to psychiatrists, two less visits to psychologists and six less visits to mental health nurses. The total cost saving for 12-months was -£1 per participant (Table 7).

### 3.5. Qualitative Results from Semi-Structured Interviews

**Figure 3:**
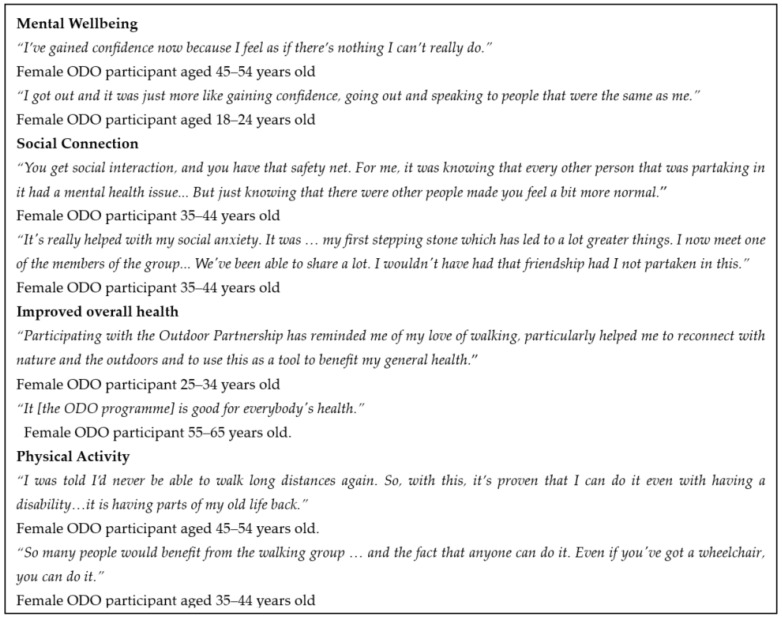
Selected quotes from ODO participants.

Supportive quotations from semi-structured interviews with ODO participants are presented in Figure 3 under the key themes: mental wellbeing, social connection, improved overall health and improved physical activity.

Mental wellbeing was the first theme developed in the analysis with participants indicating that engaging in walking improved mental health and confidence. Walking in nature was of huge value to participants and the realisation that

*‘Social interaction is actually part of my values, and it is actually needed for good mental health, and I’m an avoider by nature.’*

(Participant 3)

Participants suggested that engaging with the ODO programme was a positive intervention, particularly for individuals who were struggling to get outside and struggling with their mental health and were definitely helped as it was a supportive environment to engage in nature and with others to improve mental health by walking over the 12-week programme. In addition, being made aware from the start of the programme that it was aimed at individuals who were experiencing mental health issues made it less daunting to participate in the 12-week programme.

*‘So, it was an opportunity in a safer environment to hit my values if you like. So, to get social interaction, but have that safety net. For me, it was knowing that every other person that was partaking in it had a mental health issue. It didn’t matter if I knew what it was, but that initial realising that we’re all—we’re in different boats, but we’re in the same boat, and it’s something that nobody talks about, really, anyway. Just knowing that there were other people made you feel a bit more normal.’*

(Participant 2)

Another prominent theme to develop from the analysis of the qualitative data was social connection. Engaging with the ODO programme helped participants experiencing social anxiety and low self-esteem by providing the opportunity to create new friendships. Participants reflected that when experiencing social anxiety or psychosis, avoiding situations that require social interaction inherently made them feel safer and better but this is the wrong approach. Participants recounted that engaging with the ODO programme made them realise how important social networking is, and connecting with people and local communities was essential.

*‘We’ve been able to share a lot, I wouldn’t have had that friendship had I not partaken in this.’*

(Participant 1)

One participant recounted that when they joined the ODO programme they were not employed, and as a result the social network they previous had was gone and they missed having a social network. Engaging in the ODO programme enabled the development of a new social network and created a sense of belonging.

Improved overall health was another theme that evolved from the narrative analysis. Participants indicated that as a result of taking part in the ODO programme they were walking more and filling their down time with more productive activities outdoors than they would have undertaken before. In addition to engaging in more outdoor activity, the ODO programme inspired one participant to undertake climbing as a new hobby and motivated them to gain a qualification in rock climbing. This then sparked an interest to enrol in a couple of other courses, gain qualifications and the momentum to stay engaged with the Outdoor partnership and now is *‘giving back’* by assisting with running sessions on the ODO programme which the participant considered an important programme.

Finally, improved physical activity was the last key theme developed in the analysis. Participants suggested that they benefitted from increased levels of physical activity when involved in the guided walking session in nature. Participants indicated that walking did not make them feel body conscious compared to other activities and walking allowed you to clear your head and enjoy the scenery, while doing physical activity and as it was an enjoyable activity, encouraged retention of participants on the programme. Walking in nature allowed participants to feel normal, have a sense of belonging and acceptance, and at the end of the programme a feeling they had achieved something and had

*‘A really good dose of nature as a freebie so that helped too.’*

(Participant 5)

Overall, participants stated they experienced benefits and improvements in physical health, in terms of fitness and strength, improvement in their mental health, making new friends, confidence both socially and confidence in terms of walking, all as a result of accessing and engaging with the ODO programme.

### 3.6. Calculating the SROI Ratio

When the total social value per participant was compared with the total cost per participant, the SROI ratios ranged from £4.37 to £5.36 for every £1 invested.

### 3.7. Wellbeing Valuation Using the Social Value Bank—Sensitivity Analysis

Sensitivity analysis was conducted to generate a conservative case for the two SVB outcomes social trust, and physical activity. Sensitivity analysis allows for mitigation of study result uncertainty by exploring different real-world cases and scenarios [29].

For the conservative case, social values were awarded only to those participants who experienced an increase of 20% or more on the social trust question, and an increase of 80 min or more of walking per week (Table 8).

When deadweight, attribution and displacement were considered for the conservative case, the total social value for participants experiencing improved social trust and physical activity was £116,412 and the total social value per participant was £3085 (Table 9).

When the total social value per participant was compared with the total cost per participant for the conservative case, the social value ratio was £4.37:£1 (Table 10).

## 4. Discussion

In this social return on investment analysis of the ODO programme, the results indicated a positive social return on investment with ratios ranging from £4.90 to £5.36 for every £1 invested. Sensitivity analysis offered a conservative case scenario which generated an SROI ratio of £4.37:£1. Wellbeing valuation was applied to quantify and monetise four significant participant outcomes: mental wellbeing, physical activity, social trust and overall good health.

Although participants reported improved mental wellbeing, physical activity, social trust and overall good health, the results indicated that the use of NHS mental health services did not decrease for ODO participants during the 8-month evaluation timespan. The assessment method applied in this evaluation was a case study approach as the assessment of the ODO programme did not have a comparator intervention. The case study approach utilised in this evaluation was effective in describing, evaluating, and understanding the health and wellbeing impacts of participating in the ODO programme from a real-world context. The results are aligned with the Wellbeing of Future Generations Act (Wales) 2015 which calls for a ‘Healthier Wales’ in which people’s physical and mental wellbeing is maximised; the act also calls for a ’Resilient Wales’, where natural green space helps to support social resilience and community wellbeing. Although future studies could compare the outcomes of the walking and climbing groups, the small number of climbing participants (*n* = 11) in comparison with walking participants (*n* = 41) would likely lead to an inaccurate comparison in this evaluation. Future research examining green and natural spaces could also consider increased connection to nature as a perceived benefit to participants.

### 4.1. Strengths

Previous studies have found that outdoor walking interventions can generate positive SROI ratios and that participants reported reduced demand for health and social care services provided by the NHS and local authorities [30,31]. However, this was the first study to apply two different methods of wellbeing valuation (applying the Social Value Calculator and Mental Health Social Value Calculator), which has provided a more accurate estimation of the social value for participants who participated in a 12-week outdoor walking or climbing programme. Second, the validity of the results was strengthened from quantitative and qualitative data collected from questionnaires (*n* = 52) and interviews (*n* = 6). This mixed method approach allowed for contextualisation of participant outcomes and benefits associated with real world research. A sensitivity analysis involving a conservative case scenario was conducted to strengthen the robustness of the SROI analysis.

### 4.2. Limitations

It is acknowledged that a limitation of this evaluation is that a case study approach was applied to evaluate the health and wellbeing effects for individuals participating in the 12-week programme. It is noted that the small sample size in this real-world research may not have as effective an influence on wider populations who may have a lower propensity to engage with the programme, and its true ROI may be diluted, and this could be lower than the ROI presented within this evaluation study. It is recognised that the consistency of the results in this SROI evaluation could have may have been hindered due to the lack of a control group. Therefore, other influences (e.g., weather) were considered that could have predisposed how ODO participants completed both baseline and follow-up questionnaires. Nevertheless, this limitation was moderated by the 27% deadweight which was applied using the HACT Mental Health Social Value Calculator along with the self-reporting percentages for deadweight, attribution and displacement. In this evaluation, the research team only had access to the referral documentation and ethical approval did not include access to participants’ medical records. Therefore, the evaluation was reliant on participants’ recall of mental health service use over the period of participating in the programme.

### 4.3. Commissioning/Policy Implications

There is a need for an increased focus on prevention within the NHS and other care sectors in the UK [32]. In 2023 the UK Health and Social Care Committee announced the launch of a major new initiative calling for new research in upstream prevention to improve mental health and create healthy environments [33].

## 5. Conclusions

The importance of physical exercise in preventing avoidable morbidity and premature mortality is well accepted. Overall, our analysis showed that ODO programmes generated positive social value ratios ranging from £4.37 to £5.36 for every £1 invested. Quantitative and qualitative data from baseline and follow-up questionnaires indicated that many ODO participants improved in mental wellbeing, physical activity, social trust, and overall good health. However, ODO participants did not report a reduction in their use of NHS mental health services. Future research should measure the mental health care resource use of participants over a longer period of time, including before, during and after their ODO programme. If the results show less use of mental health services by participants during and after their programme, then it is likely that ODO will generate cost-savings to the NHS and local authorities in addition to the above social value.

## Figures and Tables

**Table 1:** Wellbeing Valuation Methods.

Outcome	Outcome Measure	Wellbeing Valuation Method
Mental wellbeing	SWEMWBS	Mental Health Social Value Calculator v.1.0
Social trust	Social trust question	Social Value Calculator v.4.0
Good health	Overall health question	Social Value Calculator v.4.0
Physical activity	IPAQ-SF	Social Value Calculator v.4.0

**Table 2:** Overview of ODO participants who completed the programme.

	ODO Participants
**Age**	96% 18–64 years-old (average 48 years old)
**Gender**	64% male, 34% female, 2% transgender
**Ethnic origin**	94% white British
**Health status**	71% of participants cited a chronic condition (e.g., anxiety, asthma, COPD, diabetes, depression, epilepsy, multiple sclerosis, psychosis, etc.)
**ODO participant group distribution**	41 walking participants, 11 climbing participants
**Employment status**	67% of participants were unemployed at baseline
	63% of participants were unemployed at follow-up

**Table 3:** Annual costs for Opening Doors to the Outdoors.

Cost Category	Total Outdoor Partnership Costs	ODO Programme Costs (17% of Outdoor Partnership Costs)
**Website (Total)**	**GBP 1608**	**GBP 273**
Website hosting	GBP 960	GBP 163
Website payment system	GBP 648	GBP 110
**Equipment and software (Total)**	**GBP 2422**	**GBP 381**
Laptop	GBP 930	GBP 32
Mobile phone	GBP 998	GBP 34
Mobile phone bill	GBP 216	GBP 37
Waterproofs and equipment	GBP 278	GBP 278
**Overheads (Total)**	**GBP 14,931**	**GBP 2538**
Company insurance	GBP 4319	GBP 734
Accounting costs	GBP 5098	GBP 867
Office costs	GBP 5514	GBP 937
**Staffing (Total)**	**GBP 71,400**	**GBP 34,023**
HWO (40% FTE)	GBP 30,600	GBP 18,558
PSO (40% FTE)	GBP 40,800	GBP 15,465
**Session (Total)**	**GBP 0**	**GBP 36,065**
Climbing admission	n/a	GBP 360
Instructor fee	n/a	GBP 35,280
Tea, coffee and refreshments	n/a	GBP 425
**Transport (Total)**	**GBP 1626**	**GBP 849**
Vehicle insurance	GBP 1136	GBP 193
Vehicle tax	GBP 490	GBP 83
Petrol/mileage	n/a	GBP 573
**Total cost per year**	**GBP 91,987**	**GBP 74,129**
**Total cost per person per year ^1^**	**n/a**	**GBP 706**

^1^ Total cost per person based on 105 ODO participants per year (three twelve-week programmes per year, 35 participants per programme).

**Table 5:** Social value outcomes adjusted for deadweight, attribution and displacement.

Outcomes	Total Social Value	Deadweight	Attribution	Displacement	Total Social Value	Total Social Value per Participants
**Social trust**	£101,331	49% (×0.51)	47% (×0.53)	17% (×0.83)	£22,734	£484 (*n* = 47)
**Good overall health**	£382,679	49% (×0.51)	47% (×0.53)	17% (×0.83)	£85,854	£2453 (*n* = 35)
**Physical activity**	£116,182	49% (×0.51)	47% (×0.53)	17% (×0.83)	£26,065	£521 (*n* = 50)
**Social impact**	**£590,921**				**£134,653**	**£3458**

**Table 6:** Social value for ODO participants using the Mental Health Social Value Calculator.

ID#	Baseline (T1)	T1 Social Value	Follow-Up (T2)	T2 Social Value	Difference (T2-T1)	Social Value	After Deadweight (27%)
1	26	£24,225	28	£24,877	2	£652	£476
2	27	£24,877	19	£17,561	−8	−£7316	−£5341
3	24	£22,944	24	£22,944	0	£0	£0
4	24	£22,944	21	£21,049	−3	−£1895	−£1383
5	7	0	19	£17,561	12	£17,561	£12,820
6	21	£21,049	21	£21,049	0	£0	£0
7	21	£21,049	26	£24,225	5	£3176	£2318
8	23	£22,944	25	£24,225	2	£1281	£935
9	16	£9639	22	£21,049	6	£11,410	£8329
10	21	£21,049	19	£17,561	−2	−£3488	−£2546
11	19	£17,561	24	£22,944	5	£5383	£3930
12	18	£12,255	20	£17,561	2	£5306	£3873
13	7	0	12	£0	5	£0	£0
14	14	0	21	£21,049	7	£21,049	£15,366
15	22	£21,049	21	£21,049	−1	£0	£0
16	7	0	7	£0	0	£0	£0
17	20	£17,561	21	£21,049	1	£3488	£2546
18	11	0	26	£24,225	15	£24,225	£17,684
20	27	£24,877	22	£21,049	−5	−£3828	−£2794
21	23	£22,944	27	£24,877	4	£1933	£1411
22	16	£9639	25	£24,225	9	£14,586	£10,648
23	20	£17,561	22	£21,049	2	£3488	£2546
24	21	£21,049	26	£24,225	5	£3176	£2318
25	18	£12,255	20	£17,561	2	£5306	£3873
26	17	£12,255	23	£22,944	6	£10,689	£7803
27	15	£9639	24	£22,944	9	£13,305	£9713
28	18	£12,255	16	£9639	−2	−£2616	−£1910
29	19	£17,561	26	£24,225	7	£6664	£4865
30	28	£24,877	25	£24,225	−3	−£652	−£476
31	22	£21,049	19	£17,561	−3	-£3488	−£2546
32	20	£17,561	19	£17,561	−1	£0	£0
33	28	£24,877	28	£24,877	0	£0	£0
34	21	£21,049	21	£21,049	0	£0	£0
35	21	£21,049	27	£24,877	6	£3828	£2794
36	22	£21,049	28	£24,877	6	£3828	£2794
37	21	£21,049	21	£21,049	0	£0	£0
38	13	£0	21	£21,049	8	£21,049	£15,366
39	16	£9639	18	£12,255	2	£2616	£1910
40	15	£9639	28	£24,877	13	£15,238	£11,124
41	21	£21,049	23	£22,944	2	£1895	£1383
42	21	£21,049	24	£22,944	3	£1895	£1383
43	30	£25,480	31	£26,175	1	£695	£507
44	10	0	24	£22,944	14	£22,944	£16,749
45	13	0	23	£22,944	10	£22,944	£16,749
46	17	£12,255	26	£24,225	9	£11,970	£8738
47	22	£21,049	24	£22,944	2	£1895	£1383
48	19	£17,561	29	£25,480	10	£7919	£5781
49	23	£22,944	28	£24,877	5	£1933	£1411
50	16	£9639	20	£17,561	4	£7922	£5783
51	24	£22,944	24	£22,944	0	£0	£0
52	20	£17,561	26	£24,225	6	£6664	£4865
**Total**		**£802,550**		**£1042,955**		**£264,630**	**£193,180**
**Total social value per participant (*n* = 51)**							**£3788**

**Table 7:** Health Service Resource Use by ODO participants.

Category	3-Months before Programme	3-Months during Programme	Difference in Visits	Cost per Visit	Cost Saving per 3-Months	Cost Saving per 12-Months
Psychiatrist	14	19	5	£51/visit ^1^	−£255	−£1020
Psychologist	38	36	−2	£58/visit ^1^	£116	£464
Mental health nurse	79	73	−6	£21/visit ^1^	£126	£504
**Total cost saving**					−**£13**	−**£52**
**Total cost saving per participant (*n* = 52)**						−**£1**

^1^ PSSRU, 2021.

**Table 8:** Conservative case outcomes adjusted for deadweight, attribution and displacement.

Outcomes:	Indicators	Quantity Improved	Financial Value	Total Social Value for Participants	Social Value per Participant
**Social Trust**	NEF Social Trust Question	18/47 reported an increase of 20% or more	£3753 per year for feeling a sense of belonging to neighbourhood	£67,554	£1437 (*n* = 47)
**Good Overall Health**	Overall health question	19/35 reported an improvement of 50% or more	£20,141 per person per year for significant improvement in good overall health	£382,679	£10,934 (*n* = 35)
**Physical Activity (Walking)**	IPAQ-SF	13/50 reported an increase of 80 min or more per week	£5281 per year for walking	£68,653	£1373 (*n* = 50)
				**£518,886**	**£13,744**

**Table 9:** Conservative case outcomes adjusted for deadweight, attribution and displacement.

Outcomes	Total Social Value	Deadweight	Attribution	Displacement	Total Social Value	Total Social Value per Participant
**Social trust**	£67,554	49% (×0.51)	47% (×0.53)	17% (×0.83)	£15,156	£322 (*n* = 47)
**Good overall health**	£382,679	49% (×0.51)	47% (×0.53)	17% (×0.83)	£85,854	£2453 (*n* = 35)
**Physical activity**	£68,653	49% (×0.51)	47% (×0.53)	17% (×0.83)	£15,402	£308 (*n* = 50)
**Social impact**	**£518,886**				**£116,412**	**£3085**

**Table 10:** SROI Ratios.

	SROI Ratio (Social Value Calculator —Conservative Case)	SROI Ratio (Social Value Calculator)	SROI Ratio (Mental Health Social Value Calculator)
Total social value per participant	£3085	£3458	£3788
NHS cost savings per participant	−£1	−£1	−£1
Total social value participant	£3084	£3457	£3787
Total cost per participant	£706	£706	£706
**SROI ratio**	**£4.37:£1**	**£4.90:£1**	**£5.36:£1**

## Data Availability

The final dataset will only be available to the study investigators and the Advisory Team. Informed consent was obtained from all subjects involved in the study.
